# Fulminant Heterotopic Ossification following COVID-19 associated Systemic Inflammatory Response Syndrome: Manifestations in Radiology, Nuclear Medicine, and Clinical Application

**DOI:** 10.1055/s-0044-1792158

**Published:** 2025-01-08

**Authors:** Deepak P. Kalbi, Edgar Zamora, Adithya Hari, Kwang J. Chun

**Affiliations:** 1 Division of Nuclear Medicine, Department of Radiology, Montefiore Medical Center, Bronx, New York, United States

**Keywords:** heterotopic ossification, joint pain and stiffness, immobility, osteoporosis

## Abstract

Heterotopic ossification (HO) is an unclear etiological trigger that results in diverse extra-skeletal bone formation in muscles and soft tissues. This often results in morbidity and reduced quality of life with pain, contractures, and mobility impairment. We present two patients with HO with a history of severe COVID-19 infection requiring 1-month-long mechanical ventilation. The first patient was found to have progressive stiffening of the right knee and left elbow, with clear demonstration of radiographic findings and active dystrophic calcification by nuclear medicine three-phase bone scan. This report might help aid earlier recognition of symptoms for an effective prevention of this debilitating disease. The other patient was also being treated with severe COVID-19, requiring intensive care unit stay with mechanical ventilation demonstrating progressive development of HO on the follow-up computed tomography (CT) images. While abdominal CT studies were obtained for this patient to evaluate the focal sites for infection, the patient was too ill and unable to undergo a bone scan study.

## Introduction

Heterotopic ossification (HO) is a condition with an unclear cause that leads to abnormal bone growth in muscles and soft tissues. This can result in pain, joint stiffness, and difficulty with movement, ultimately affecting a person's quality of life. 1 2 3 HO is defined as the abnormal formation of lamellar bone in soft tissues, often containing bone marrow. It has been associated with musculoskeletal trauma, surgery, burns, neurologic injury, immobilization, and congenital and metabolic disorders. 4 5 More recently, it has also been reported as one of the sequelae of critical illness. 6 7

## Presentation of Cases

**Fig. 1:**
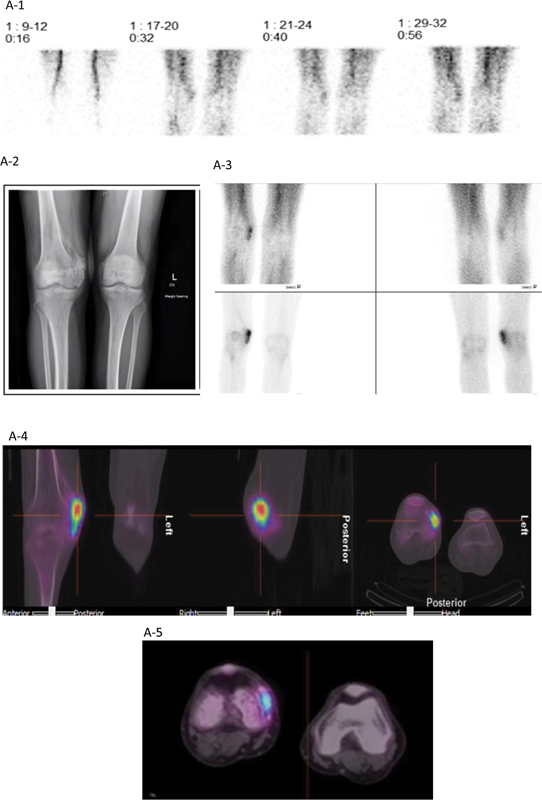
( **A-1** ) First phase of the three-phase bone scan. ( **A-2** ) Radiograph of the right knee showed bone formation in the medial condyle and medial supracondylar cortex of the right femur. ( **A-3** ) The second phase (upper panels) and third phase (lower panels) of the three-phase bone scan. ( **A-4** ) Single photon emission tomography with computed tomography (SPECT-CT) of the area of interest showed increased radiotracer uptake near the medial epicondyle of the right femur. Distal right femur showed an osteoporotic change compared with the left distal femur on the low-dose CT axial image. Bone scan was obtained 7 months after initial COVID-19 infection with severe respiratory illness. ( **A-5** ) Magnified SPECT-CT image of the knees. ( **B-1** ) X-ray images of the patient's left elbow showed mature bone formation in the distal left humerus and presurgical evaluation of 3D CT left elbow (posterior view) revealed similar findings. ( **B-2** ) SPECT-CT of the left elbow bone scan demonstrates active heterotopic ossification. ( **C-1** ) Planar anterior and posterior whole body bone images. Radiotracer dose administered intravenously in the right arm. ( **C-2** ) SPECT-CT of the pelvis (10 months post-ICU stay with mechanical ventilation) and the patient indicated hip pain.

**Fig. 2:**
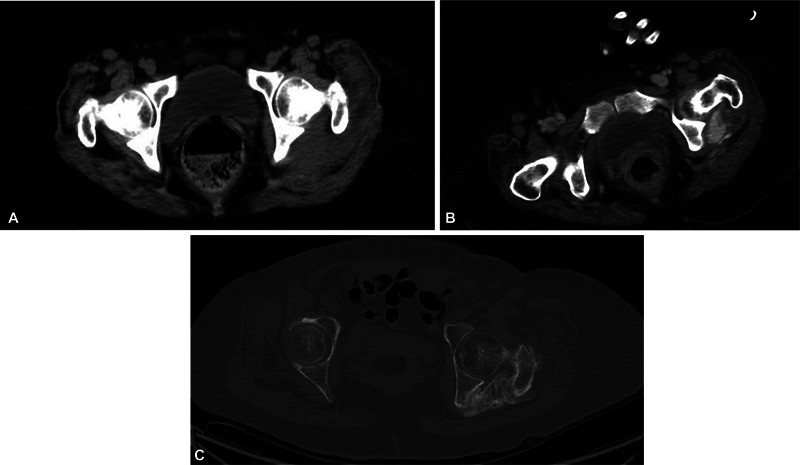
( **A** ) Very early stage, cross-sectional computed tomography (CT) image of the pelvis. ( **B** ) Nine days later, there is development of visible sclerosis in the left buttock without touching the neighboring bones. ( **C** ) Seven months later, CT image of the pelvis showing soft tissue sclerosis (heterotopic ossification), advanced stage, demonstrating bony ankylosis between the proximal left femur and pelvic bone.

A 66-year-old man with history of severe COVID-19 infection requiring mechanical ventilation for 1 month developed pain and stiffness of his right knee and left elbow after being discharged to the rehabilitation facility. When he presented for bone scan, he denied fever, chills, night sweats, changes in weight, or any other past medical problems except for the COVID-19 infection incident. Focused physical examination revealed right knee medial-sided bony mass with limited range of motion, but otherwise neurovascularly intact. The medial aspect of the left elbow also revealed palpable hard mass with tenderness, joint stiffness, and limited range of motion. For preoperative plan of joint surgeries, a three-phase bone scintigraphy was performed using radiopharmaceutical technetium-99m-methylenediphosphonate ( ^99m^ Tc-MDP). The phases of bone scan—the immediate vascular perfusion phase, the intermediate blood pool activity, and the delayed radiotracer uptake in the bone—are noted to be increased in HO, denoting the active dystrophic calcification process. Fig. 1A-1 to A-5 shows the beginning of hypervascularity in the medial aspect of the right knee. The three-phase bone scan performed with 740 MBq (20 mCi) of ^99m^ Tc-MDP showed increased vascularity, hyperemia, and delayed image with increased tracer uptake in the medial aspect of right knee. The elbow X-ray and CT of the elbow are shown in Fig. 1B-1 and B-2 . Multifocal HO involving the left elbow, bilateral hips, and right knee is shown in Fig. 1C-1, C-2 .

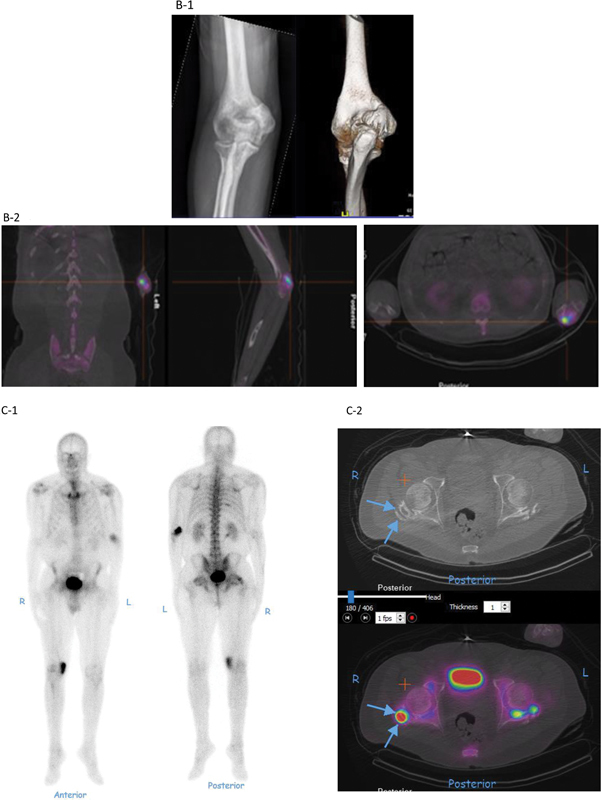



The second patient is a 57-year-old woman with COVID-19 who developed acute inflammatory demyelinating polyradiculopathy/Guillain–Barre syndrome, 8 9 progressing from headache and ataxia to quadriparesis. The pelvic CT scans at three different time points (early January, mid-January, and then July, all in the same year, 2021) demonstrated HO at three different stages of progression, starting with nearly negligible sclerotic changes in the soft tissue of the left buttock ( Fig. 2A ), progressing to visible sclerosis ( Fig. 2B ), and, finally, expansion of HO, touching neighboring bones ( Fig. 2C ).

## Discussion

HO is clinically identified by pain, swelling, and progressive stiffening of the affected site. The pathophysiology of HO includes a cascade of stimulation of local and systemic factors that induce pathologic recruitment and differentiation of osteoprogenitor cells and further proliferation of osteoblasts. The etiology of HO is multifactorial, which includes, but is not limited to, trauma, neurological insult, tissue hypoxia, and hypermetabolic status. 10 11 Calcium homeostasis is reported to be perturbed. Very few case reports implicate critical illness like COVID-19 infection as the inciting factor of HO. 12 13 Complications of HO include severe morbidity, pressure ulcers, and peripheral nerve entrapments. Hence, early diagnosis of HO is extremely important. Three-phase bone scintigraphy is the most sensitive imaging modality for the diagnosis of HO. This coupled with the use of single-photon emission computed tomography with computed tomography (SPECT-CT) further increases the diagnostic accuracy of the three-phase bone scan in identifying active disease. This modality not only helps in early diagnosis of HO but also identifies the active dystrophic calcification process. 14 Early diagnosis of HO helps direct therapy toward preventing the formation of HO through rigorous physical therapy and the use of nonsteroidal anti-inflammatories. Identification of the ongoing pathophysiological process in the patient helps the clinician to avoid choosing a surgical approach as a treatment option. Surgical resection is usually delayed till HO achieves maturity to decrease intraoperative hemorrhage and postoperative recurrence. COVID-19 infection is associated with thrombogenic tendencies, causing coagulopathy prone to develop local tissue damage. Further surgical intervention can be helped in the area for minimizing postarthroplasty complications utilizing a comprehensive approach that considers preoperative optimization, including preoperative densitometry, optimization of bone quality through at least 3 months of bone-strengthening medications if low bone mineral density is found, and early diagnosis of HO decreasing surgical complications can improve patient outcomes, reduce health care cost, and enhance patient satisfaction.
